# Challenges in professional development of anaesthesiology trainees: A cross-sectional survey by the European Society of Anaesthesiology and Intensive Care Trainee Committee

**DOI:** 10.1097/EA9.0000000000000062

**Published:** 2024-09-20

**Authors:** Olivia Dow, Antonia Kustura, Yotam Lior, Joana Berger-Estilita, Diogo Morais, Stefan De Hert, Igor Abramovich

**Affiliations:** From the Royal Surrey NHS Foundation Trust, Guildford, UK (OD), Division of Anaesthesiology, Intensive Care and Pain Medicine, Sestre milosrdnice University Hospital Centre, Zagreb, Croatia (AK), Division of Anaesthesia, Intensive Care, and Pain Management, Tel Aviv Medical Centre, Faculty of Medicine, Tel-Aviv University, Tel-Aviv, Israel (YL), Institute for Medical Education, University of Bern (JB-E), Institute of Anaesthesiology and Intensive Care, Salemspital, Hirslanden Medical Group, Bern, Switzerland (JB-E), CINTESIS@RISE, Centre for Health Technology and Services Research, Faculty of Medicine, University of Porto, Porto, Portugal (JB-E), Department of Anaesthesiology, Centro Hospitalar De Trás-Os-Montes E Alto Douro, Villa Real, Portugal (DM), Department of Anaesthesiology and Peri-operative Medicine, Ghent University Hospital (SDH), Department of Basic and Applied Sciences, Ghent University, Ghent, Belgium (SDH) and Department of Anaesthesiology and Intensive Care Medicine (CCM, CVK), Charité – Universitätsmedizin Berlin, Corporate Member of Freie Universität Berlin and Humboldt Universität zu Berlin and Berlin Institute of Health, Berlin, Germany (IA)

## Abstract

**BACKGROUND:**

The coronavirus disease (COVID-19) pandemic disrupted training in anaesthesiology. The global shortage of healthcare workers has also negatively affected training opportunities.

**OBJECTIVE:**

To evaluate the current experiences, challenges and professional development of anaesthesiology trainees across Europe.

**DESIGN:**

An online cross-sectional survey of anaesthesiology trainees.

**PARTICIPANTS:**

Anaesthesiology trainees from the 42 European Society of Anaesthesiology and Intensive Care (ESAIC)-affiliated countries.

**MAIN OUTCOME MEASURES:**

Quality of training supervision and program structure; the impact of COVID-19 on training and practical learning, trainee support systems, financial challenges and professional development, involvement in the ESAIC Exchange Program and career mobility insights.

**RESULTS:**

Seven hundred and seventy-one participants from 35 ESAIC-affiliated European countries highlighted several aspects of anaesthesiology training. Approximately 30.5% of trainees reported being able to independently anaesthetise patients with ASA class 1 and 2 within 3 months, whereas independence for ASA 3 and 4 patients under indirect supervision was achieved by 24.3% between 6 and 9 months. Half of the respondents attained an independent working capacity in the ICU after 1.5 years. Although 51.3% reported receiving adequate training and supervision, only 30.1% received employer financial support for educational activities. The COVID-19 pandemic led to 40.2% being redeployed, primarily to ICUs, with 45.2% experiencing negative training effects. Career-wise, 12.5% engaged in international exchange programs, and 49.1% considered relocating for better career opportunities and work–life balance.

**CONCLUSION:**

The findings provide valuable insights into the current state of anaesthesiology training in Europe, highlighting the need for adaptive strategies in medical education and training to meet evolving challenges and ensure continual professional growth.

**KEY POINTS**

- About 30.5% of trainees reported the ability to independently anaesthetise ASA 1 and 2 patients within the first 3 months, 24.3% achieved indirect supervision independence for ASA 3 and 4 patients between 6 and 9 months and 50.4% attained independent working capacity in the ICU with senior support after 1.5 years.
- The COVID-19 pandemic had a significant negative effect on training, with 40.2% of respondents redeployed, mainly to ICUs, and 45.2% reported negative effects on their training. However, a significant portion (43.5%) did not perceive the need to compensate for lost time.
- Participation in international exchange programs was reported by 12.5% of the respondents, primarily for skill development and gaining life experience. The main barriers to participation included cost, family constraints and lack of local authorisation.

## Introduction

Anaesthesiology encompasses a broad scope of medical practice including peri-operative, emergency, critical care and pain management. Consequently, training in anaesthesiology is challenging because of the broad scope of knowledge and skills required, accompanied by lengthy training and assessment via difficult examinations.^1,2^ Training and effective supervision are therefore crucial for a resident's success, with evidence linking better supervision to improved outcomes.^3^

Training approaches in anaesthesiology have evolved towards competency-based methods,^4^ with organisations, such as the European Union of Medical Specialists (UEMS), the European Board of Anaesthesiology (EBA) and the European Society for Anaesthesiology and Intensive Care (ESAIC) continually updating standards for harmonised European training.^5^ Challenges like the global healthcare worker shortage, exemplified by the European Working Time Directive's effect on educational opportunities, add complexity to existing anaesthesiology training landscapes.^6,7^

In the UK, the General Medical Council (GMC) conducts annual surveys assessing training programs, revealing issues such as burnout and the need for better support and workplace culture.^8–10^ Despite this, there is a lack of such data outside the UK, often leaving trainees dependent on departmental programs or grant funding for education. This disparity in training conditions across Europe has sparked concerns regarding trainee emigration.

In 2015, we conducted a comprehensive survey^11^ to assess the state of anaesthesiology and intensive care training at that time. The European Society of Anaesthesiology and Intensive Care Trainee Committee (ESAICTC) has previously identified education, financial issues, and career prospects as major concerns among trainees. The goal of this updated survey was to review the quality of training and how it differs across European programs, as well as overall satisfaction with anaesthesiology training. This survey aimed to assess the current state of anaesthesiology training in Europe, examining aspects such as trainee supervision, workload and healthcare worker shortages. Notably, the COVID-19 pandemic has caused significant disruptions, with reduced elective surgery and redeployment of trainees to critical care units particularly affecting educational opportunities.^12^ Additionally, the survey addressed issues such as financial support for training and the risk of burnout among trainees. The objective was to identify the main concerns of European anaesthesiology trainees, such as education quality, financial burdens and career prospects, and to assess their satisfaction with their training programs. This information is crucial for decision-makers and policymakers to understand and improve the training environment and career outlook of European anaesthesiology trainees.

## Methods

### Ethics

Ethics approval for this cross-sectional online survey-based study (BASEC-Nr: Req-2023-00634) was waived by the Cantonal Ethics Committee of Bern (KEK Bern, Murtenstrasse 31, Hörsaaltrakt Pathologie, Eingang 43A, Büro H372, 3010 Bern, Switzerland, 15 May 2023). The respondents were asked to provide consent for participation and data analysis

No identifying data were collected to ensure participant anonymity, and all survey data were securely stored in a repository accessible only to the research team. The study complied with the Declaration of Helsinki^13^ and the Data Protection Acts of the researchers’ centres.

### Survey outcomes

We determined the primary and secondary outcomes based on the significance and direct impact of the surveyed areas on anaesthesiology training in Europe.

Primary outcomes:

(1) Training conditions and supervision: this includes aspects such as the adequacy of supervision, the variety and complexity of cases handled and the overall structure of the training program.
(2) COVID-19 pandemic effects on training: including changes in training schedules, reduction in elective procedures, shift to emergency and critical care duties and the overall effect on hands-on training opportunities and learning objectives.

Secondary outcomes:

(1) Support during training including aspects of burnout, access to psychological support and financial constraints that trainees face, which can indirectly affect their learning and professional development.
(2) Participation in exchange programs and migration prospects: the involvement in exchange programs and views on migration for work or training provides insight into anaesthesiology trainees’ broader career aspirations and mobility.

### Survey design

The survey (Appendix 1) was designed to align with the objectives above. This design was based on the precedent established in the 2018 survey iteration, which targeted trainees in anaesthesiology and intensive care across Europe. The current iteration incorporated modifications to address challenges and developments, notably the impact of the COVID-19 pandemic. The survey comprised 37 items written in English, designed to gather voluntary responses from a diverse sample of European anaesthesiology trainees. Among these, 34 items were structured as multiple-choice questions supplemented by optional free-text responses to facilitate more nuanced expressions by participants. The remaining three items were designated as ranking questions. A critical exclusion criterion was that participants who had completed their training for more than 12 months before the commencement of the survey were automatically directed to conclude their participation. To contextualise the responses, we also collected details such as the year of training, length of training, country of work and ESAIC membership status. Data collected included respondents’ training year, training duration, country of practice and ESAIC membership status, providing an essential context for the analysis of the responses.

Before deployment, the questionnaire underwent a pilot test, involving 10 clinicians from seven countries and two experts in medical and qualitative research. The survey, which took an estimated 10 minutes to complete and was spread over 18 pages, allowing participants to pause, resume and revise their responses before the final submission.

### Data collection period

The trainee survey was conducted over 6 months, from June to November 2023. The methodology for distributing the survey primarily involves the ESAIC's network. The survey was disseminated through ESAIC's channels and its Trainee Network's social media platforms, which are frequently visited by healthcare professionals in the field of anaesthesiology. Additionally, the survey link was shared via national anaesthesiology societies across Europe to broaden its reach.

The respondents could freely answer questions in a fixed order. The survey used branching, where answers to questions about congress attendance and migration triggered follow-up questions for deeper insights into the specific interests and concerns of European anaesthesiology trainees.

To facilitate easy access and participation, the survey was hosted on the SurveyMonkey platform, a server-based system allowing respondents to participate through internet browsers and smartphones, eliminating the need for additional software. There was no direct contact with individual trainees; instead, anyone accessing the survey link through the ESAIC Trainee Network or their respective national societies was eligible to respond.

The ESAIC promoted the survey at the annual Euroanaesthesia Congress 2023 in Glasgow and endorsed the survey via E-Mail in August 2023.

Up to two reminders were sent to potential respondents. Similar to a previous study,^11^ we aimed to collect at least 500 responses from all trainees in ESAIC-affiliated countries.

### Statistical analysis

The survey data was analysed and treated per Data Protection Regulations, ensuring that the information remained anonymous. The results were imported automatically into a password-protected databank and analysed using SPSS software. The responses were clustered according to the United Nations Geoscheme.^14^ Descriptive statistics were employed to summarise the survey data, presenting categorical variables as counts (*n*) and proportions (%) and continuous variables as mean ± SD or median [IQR, range] as appropriate. The normality of individual survey items was assessed using the Shapiro–Wilks test, residual visualisation and Q-Q plots. Parametric data were analysed using Student's *t* test and Pearson's *χ*^2^ statistics for contingency table analyses (two-sided). Significance was set at *P* less than 0.05. Statistical analyses were performed using SPSS Statistics version 27 (IBM Inc., New York, USA).

## Results

### Respondents’ characteristics

**Fig. 1:**
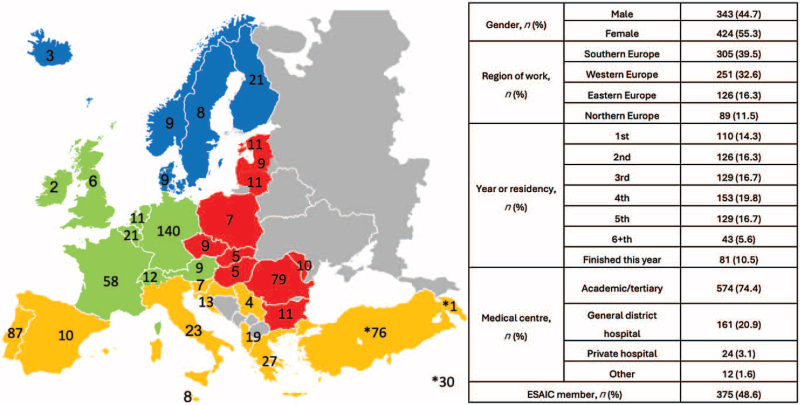
Respondent's characteristics.

**Fig. 2:**
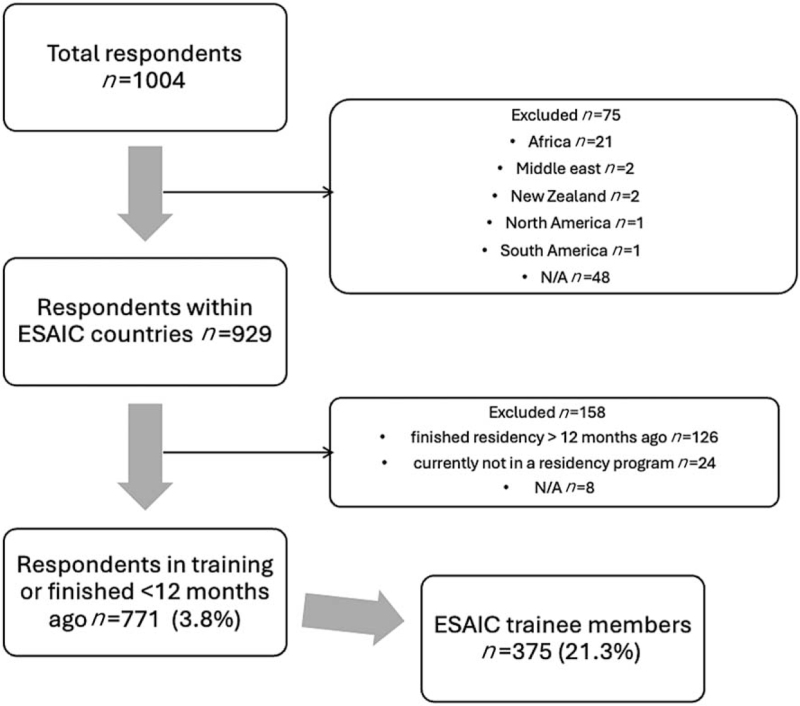
The flowchart outlines the selection process of respondents for the study.

A total of 1004 anaesthesiology residents participated in the survey. Figure 1 shows the respondents’ characteristics. Excluding responders from non-European countries and anaesthesiologists who had completed training 12 months before the survey, 771 trainees responded to the survey, resulting in a response rate of 3.8% based on an estimated number of anaesthesiology trainees in Europe of about 20 082.^15^ Of the 771 included responders, 375 declared themselves to be trainee members of ESAIC, reflecting a 21.3% response rate of the total registered 1761 trainee members in 2023 (Fig. 2).

The diagram illustrates the geographic distribution and demography of survey respondents in Europe from 35 ESAIC affiliated countries. The map delineates regions by colour according to the United Nations Geoscheme^13^ (blue – North Europe; red – East Europe; yellow – South Europe; green – West Europe), with numerical annotations indicating participant counts. Israel, Turkey and Armenia were included in the south Europe group. The accompanying table specifies respondent characteristics in numbers and percentages.

Nonmembers cited cost (42.1%), lack of perceived benefits (28.1%) and membership of other societies (40.3%) as reasons for not joining ESAIC. The survey data revealed that 87 out of 615 respondents attended Euroanaesthesia 2023, accounting for a 14.1% attendance rate. The key reasons for attendance included presenting scientific work (48.3%), gaining knowledge (75.9%), networking (69%) and exploring new technologies (32.2%). When asked about support for Euroanaesthesia 2023, 5 of 87 (5.7%) responders stated that all the expenses were covered by the hospital, 15 of 87 (17.2%) stated that the hospital covered only the registration fee, and 62 of 87 (71.3%) were self-funded. Free-text responses indicated that trainees did not attend Euroanaesthesia 2023 because they were not aware of the conference.

When asked about what should be prioritised by the ESAIC to support trainees, respondents ranked resident wellbeing, median = 3 [1 to 4] as the most critical target to address. This was followed by employment prospects, exchange program and workload and supervision, median = 3 [2 to 4]. Exam/Educational support, median = 4 [3 to 5], was considered to be the lowest priority.

### Training conditions and supervision

**Fig. 3:**
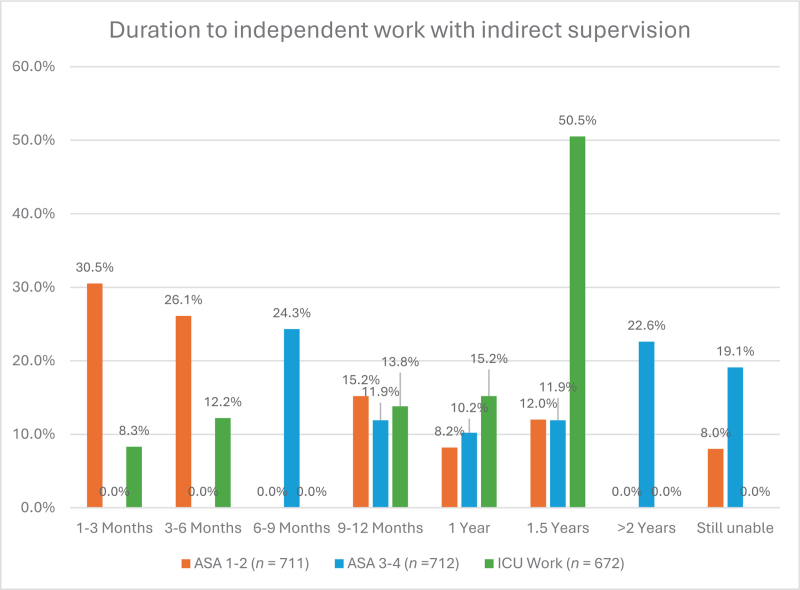
The bar chart quantifies the time required for anaesthesiology trainees to reach proficiency in conducting independent procedures under indirect supervision, stratified by patient complexity: ASA classifications 1 and 2 (*n* = 711) and 3 and 4 (*n* = 712), and in an ICU setting (*n* = 672).

Figure 3 consolidates the trainees’ responses on achieving autonomy in anaesthetising patients classified as ASA 1 and 2, ASA 3 and 4, and independently working in the intensive care setting, all under indirect supervision.

**Fig. 4:**
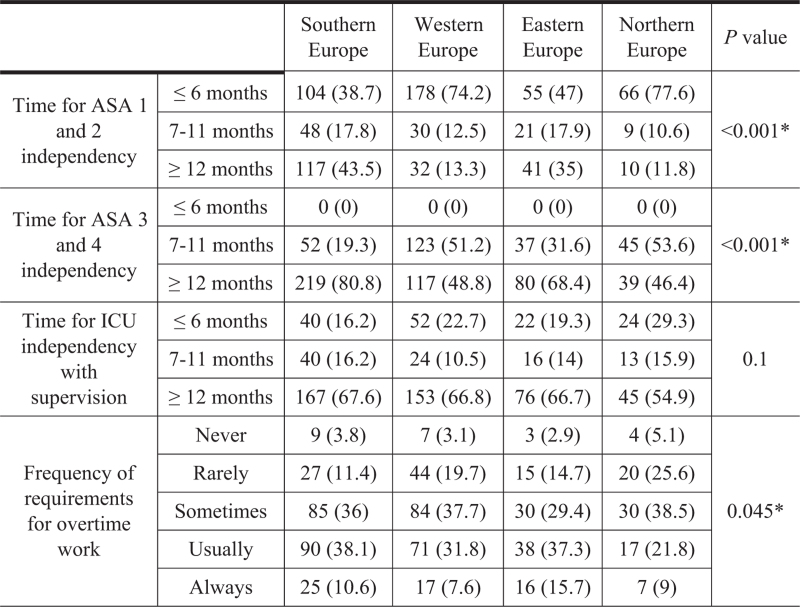
Geographic variations in training proficiency and overtime requirements.

Trainees from Northern and Western Europe reported achieving independence in administering anaesthesia to ASA 1 and 2 patients at 6 months, earlier than their counterparts in Southern and Eastern Europe. This difference was statistically significant (*P* < 0.01). For ASA 3 and 4 patients, trainees in Northern and Western Europe attained independent practice within 6 to 12 months, sooner than those in Southern and Eastern Europe who achieved autonomy after more than 12 months (*P* < 0.01). Independence in the ICU was fairly uniform, with most respondents reporting a duration of more than 12 months (*P* = 0.1; Fig. 4).

This figure presents the distribution of training stages and overtime work across various regions, quantified using the *χ*^2^ Test. Data are given as *n* (%). Percentages are calculated relative to the total number of responses within each region for every category. ^∗^Statistical significance is denoted at the *P* < 0.05 level.

Of the 639 respondents, 328 (51.3%) subjectively felt that they received an adequate amount of training and supervision, 245 (38.3%) agreed and 83 (13%) strongly agreed with the statement. In addition, 41.9% (265/633) met, 37.1% (235/633) exceeded, and 5.5% (35/633) fell significantly short of the required number of practical operations or procedures for their training level.

The median number of hours dedicated to clinical work with all commitments per week was 53 h [47 to 61]. Of the respondents, 10.2% (65/639) reported ’always’ working beyond their rostered hours. Most indicated working extra hours, 'sometimes’ (35.8%, 229/639) or ’usually’ (33.8%, 216/639). Only 3.6% (23/639) reported ’never’ being required to work additional hours. Respondents from North Europe report less frequent overtime requirements compared with other regions (*P* = 0.045) (Fig. 4).

Half of the respondents (50.4%, 322/639) assessed their current workload as ’about right’, while a near-equal proportion (44.8%, 286/639) described it as ’excessive’. A minority (4.9%, 31/639) perceived their workload as insufficient. Compared with the pre-pandemic area, most (38.3%, 245/639) participants reported no significant changes, whereas 14.4% (92/639) experienced a notable increase in workload.

Factors contributing to the workload (*n* = 613), by order of perceived significance, included: on-call shifts, median = 1 [1 to 2], extra shifts, median = 2 [2 to 3], examinations, median = 3 [3 to 4], and postgraduate qualifications or scientific work, median = 4 [3 to 5].

### Coronavirus disease 2019 pandemic effects on training

During the COVID-19 pandemic, 40.2% (169/420) of the respondents were redeployed. Notably, 48.9% (205/419) were re-assigned to intensive care units. Approximately half of the respondents (45.2%, 189/418) reported that the pandemic adversely affected their training time and practical opportunities. Conversely, 43.5% (182/418) did not perceive a need to compensate for lost time, 33% (138/418) lacked opportunities to compensate for this deficit and 23.4% (98/417) were provided with opportunities to recover lost training time. Only a minority (11.7%, 49/419), indicated the necessity of extending their residency program because of the pandemic's impact. Of note, two-fifths of the respondents (40.7%, 260/639) began their residency program during or after the pandemic.

### Support during training

Regarding trainees’ wellbeing, 44.2% (269/608) reported receiving adequate support in their workplaces. Approximately 16.6% (101/608) had access to coaching services and 17.6% (107/608) reported being provided with psychological assistance. One-third (33.4%, 203/608) had access to mentoring. Access to nonmedical support (10.3%, 63/608) and counselling (12.2%, 74/608) were less common.

When questioned about the impact of financial constraints in various activities, by order of perceived significance, 613 respondents mentioned limitations in conference attendance, median = 2 [2 to 3], enrolment in courses, median = 3 [2 to 3], access to books and journals, median = 3 [2 to 4] and membership of scientific societies, median = 3 [2 to 4].

One-third of the respondents (30.1%, 182/605) reported a lack of financial support for educational activities: 24.1% (146/605) received partial support, whereas 20.3% (123/605) and 19.7% (119/605) received occasional or rare financial support, respectively. A minority (5.6%, 34/605) reported full financial support from their employers.

### Participation in exchange programs and migration prospects

A small number of respondents (12.5%, 76/606) reported participation in international exchange programs outside of their home country. The primary motivations for participation were skill development at a renowned institution (69.7%, 53/76), life experiences and exposure to new cultures (67.1%, 51/76) and participation because of institutional requests (7.9%, 6/76). Additional reasons included pursuing job opportunities and engaging in a research fellowship.

Responses from 519 respondents provided key reasons for not participating in exchange programs: costs (38.5%, 200/519), family constraints (35.5%, 184/519), lack of local authorisation (24.1%, 125/519) and disinterest (13.9%, 72/519). Other less common reasons included plans for later participation and need for recognition during training.

Nearly half of the respondents contemplated relocation (49.1%, 297/605). The primary motivations identified were improved work–life balance (63.3%, 188/297), broader career opportunities (51.9%, 154/297), economic factors (51.9%, 154/297) and gaining life experience (51.9%, 154/297). Less common reasons included intention to return to their home country (5.1%, 15/297) and family-related factors (8.8%, 26/297). Other notable reasons are related to the political and social climate.

**Fig. 5:**
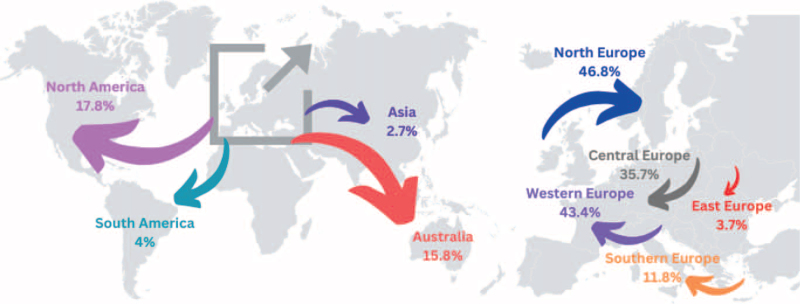
The map provides a detailed visualisation of anaesthesiology trainees’ preferred migration patterns within Europe and globally (*n* = 297).

Preferred regions for immigration are depicted in Fig. 5.

Northern Europe emerges as the most favoured, attracting 46.8%, followed closely by Western Europe at 43.4%, while smaller proportions prefer to relocate to other areas such as North America.

## Discussion

In this survey, we assessed the experiences, challenges and professional growth of European anaesthesiology trainees. Many respondents achieved independence in anaesthetising ASA 1 and 2 patients within 3 to 6 months, but there was a variation across regions, with trainees in Northern and Western Europe reporting earlier independence. The pandemic led to the redeployment of many trainees, particularly to intensive care units, with nearly half reporting a negative impact on their training time and practical opportunities. Less than half of the respondents felt that they had received adequate workplace support. Only a minority of trainees participated in international exchange programs, primarily because of financial and family constraints. However, nearly half of the respondents considered migration for better work–life balance and career opportunities, showing a trend towards high mobility among trainees.

### Independence in anaesthesia practice

The survey data reflect significant variability in the independence and supervision levels experienced by anaesthesiology trainees across Europe. Heterogeneity in residency experiences may be rooted in the varied organisational structures of the training programs. Some regions have more flexible curricula, leading to early autonomy for trainees, whereas others maintain competency-based frameworks that necessitate prolonged direct supervision.^16^ These differences might also extend to the distribution of weekly duty hours, contributing further to disparate training experiences.^17^

The rate at which trainees achieve autonomy may also correlate with the availability of training resources, including simulation-based learning.^18^ Northern and Western Europe's rapid autonomy attainment may be attributed to widespread access to such educational tools, which allow risk-free skill development. In contrast, the slower progress in Eastern and Southern Europe may reflect less access to these advanced training resources, suggesting a need for an improved distribution of simulation training across Europe.^19^

Although there is a general trend towards achieving independent practice in the ICU setting after 1.5 years, the consistency across regions may be because of the nature of ICU work. Given the complexities of caring for critically ill patients, a high level of proficiency and wide array of competencies are required.^20^ This is underscored by the fact that ICU rotations typically last at least 12 months in most countries.^16^ In addition, large teams and senior support are more common in the ICU setting, promoting an environment with more psychological safety for learning.^21^ Finally, the standardisation of intensive care training across Europe, often as a subspecialty with a 2-year duration, might contribute to a more uniform experience in acquiring autonomy in ICU rotations.^22^

### Effects of coronavirus disease 2019

The survey revealed that the COVID-19 pandemic significantly affected training programs, with over 40% of respondents being re-assigned mostly to ICUs, and nearly half having negative effects on their residency training. This phenomenon probably occurred on a global scale and has been described in several studies.^10,23,24^ Despite these disruptions, many did not feel the need to compensate for the lost training time, indicating an effective adaptation to the challenges brought by the pandemic. Examples of such adaptations include increasing online educational offerings and the implementation of curricular flexibility.^23^ It also indicates that the experience gained in ICUs during redeployment may have provided a comprehensive and valuable learning experience. The ICU environment often demands a wide range of medical skills and knowledge, possibly contributing to more complete training in various areas, thus compensating for other aspects of training that were disrupted due to the pandemic.^25^

### Support in the workplace

In the aftermath of the pandemic, there has been a notable increase in the impact on mental health, contributing to an elevated burnout rate.^26^ This is particularly concerning in anaesthesiology, where burnout rates are among the highest across all medical professions.^27^ The need for support mechanisms for trainees is aligned with the increasing mental health challenges and reports of most anaesthesiologists receiving little to no training on coping with stressful situations, such as night shifts.^28^ Our findings reinforce a critical gap: the need for a cultural shift towards more supportive work environments in anaesthesiology. There is a clear demand for education and training that promotes safe practices and fosters a culture of support, acknowledging and addressing the mental and emotional demands placed on medical professionals.^29^

In line with our findings, several surveys document the financial challenges of trainees attending educational activities and congresses and the higher costs associated with engaging in research activities.^30–32^ To support trainees in extra-curricular education and congress attendance, establishing dedicated budgets could be beneficial. This approach has already been adopted in some programs in the United States of America and in the United Kingdom, where residents receive specific funding for such activities.^33^

### Trainee mobility

Our findings indicate a notable trend for relocation for career advancement among trainees, with 49.1% looking towards North, West and Central Europe. International exchange programs, essential for skill enhancement and life experiences in connected Europe, saw limited engagement from our respondents, contradicting the UEMS EBA's push for increased migration.^34^ Improved awareness and recognition for accreditation of these programs could enhance participation, meeting the goals of enriching experiences and aiding migration. Notably, many respondents highlighted financial support as a primary issue, underscoring the need for improved funding mechanisms to alleviate costs associated with these programs. Alternatively, integrating such programs into residency rotations, as practised by some European centres, could improve future mobility and accessibility for all trainees.^35,36^ Additionally, there may be a need to examine and improve residency and working conditions in less attractive regions to maintain the appeal, and to prevent a medical “brain drain” similar to what was observed in Ireland in past decades.^37^

Our study encompasses 3.8% of all European Trainees, larger than those in previous methodological studies, providing a better representation of ESAIC trainee members.^11^ However, this may not fully represent the entire demographic data. The use of self-reported data in our survey introduces the possibility of bias, as responses might be influenced by individual perceptions, particularly regarding the aspect of migration. This bias is heightened by the survey's distribution among ESAIC members, a factor that could shape personal viewpoints and responses. Geographical and cultural diversity within Europe, along with the unique impacts of the COVID-19 pandemic, could also affect the generalisability of our findings. Acknowledging these limitations is crucial for a nuanced interpretation of the results and highlights areas for future research.

## Conclusion

Our survey highlighted a significant variation in training experiences, marked by differences in autonomy levels and geographical disparities. This also underscores the critical need for adequate psychological support and wellbeing initiatives for trainees. Furthermore, financial constraints appear to be a notable barrier to educational opportunities and international exchange programmes. These findings suggest that room for improvement remains, particularly in supporting trainee wellbeing and ensuring equitable access to educational resources. As the medical field of anaesthesiology continues to evolve, especially in the wake of the pandemic, these insights are valuable for shaping future training programs and policies to better address the needs of anaesthesiology trainees.

## Supplementary Material

Supplemental Digital Content
