# Supplementary material for: Challenges in professional development of anaesthesiology trainees: A cross-sectional survey by the European Society of Anaesthesiology and Intensive Care Trainee Committee
Source: Eur J Anaesthesiol Intensive Care. 2024 Sep 20;3(5):e0062. doi: 10.1097/EA9.0000000000000062 (PMC11798376; doi:10.1097/EA9.0000000000000062)
Supplement: Supplemental Digital Content [file ejaic-3-e0062-s001.pdf]

Introduction

This survey, supported by ESAIC Trainee Network, aims to assess the quality and differences in anaesthetic training across Europe. Its purpose is to identify the primary issues and overall satisfaction of trainees. We will compare our results with a similar study conducted in 2015 to determine how to improve the quality and status of residency programs.

By completing this questionnaire, you grant us permission to anonymous publication of your responses. The survey is online, takes about 5-8 minutes to complete, and is entirely voluntary. Your responses will be kept anonymous and confidential.

The Bernese Cantonal Ethical Committee, Switzerland (BASEC-Nr: Req-2023-00634, 15/05/2023) waived this research project's ethical approval.

We appreciate your participation and encourage you to share the survey link with your colleagues.

Antonia and Olivia from the ESAIC Trainee Network

DEMOGRAPHICS

1. Which country do you work in?

2. What year of residency are you in?

- 1st
- 2nd
- 3rd
- 4th
- 5th
- 6th year and beyond
- I finished my residency in the last 12 months
- I finished my residency over 12 months ago
- I am currently not in a residency program

3. What is your gender?

- Female
- Male
- I prefer not to say

4. What type of centre do you work in?

- Academic/ tertiary centre
- General district hospital
- Private hospital
- Other (please specify)

5. Are you a member of the European society of Anaesthesiology and Intensive care (ESAIC) ?

- Yes
- No

6. If you are not a member, would you like to share why?

- [ ] Cost
- [ ] I didn't think I would benefit from being a member
- [ ] I am already part of other anaesthesiology societies
- [ ] Other (please specify)

AUTONOMY TRANSITION

7. At which stage during your training were you able to **independently anaesthetise non-complex/ ASA 1-2 patients** in the **operating room** with **indirect supervision** of a senior colleague within the department?

- ◯ 1-3 months
- ◯ 3-6 months
- ◯ 9-12 months
- ◯ 1 year
- ◯ 1 ½ years
- ◯ I am still not able

8. At which stage during your training were you able to **independently anaesthetise complex/ ASA > 3** in the **operating room** with **indirect supervision** of a senior colleague within the department?

- ◯ 6-9 months
- ◯ 9-12 months
- ◯ 1 year
- ◯ 1 1/2 years
- ◯ After >2 years
- ◯ I am still not able

9. At which stage were you able to **work independently** in the **intensive care unit (ICU)** with **senior support in the background**?

- ◯ 1-3 months
- ◯ 3-6 months
- ◯ 9-12 months
- ◯ 1 year
- ◯ after 1 ½ years

TRAINING AND WORKLOAD

10. Have you completed, or are you on track to complete, the necessary amount of practical operations or procedures required for your current level of training?

- Yes - more than the required number
- Yes - about the required number
- No - not quite the required number
- No - significantly below the required number

11. What is the **average number of hours per week** you dedicate to **clinical duties** (including regular and on-call commitments)?

Please move the slider to the amount of hours per week

0 40 100

12. How would you assess the amount of work you have at the moment?

- Excessive
- About right
- Could be more

13. How does your current workload compare to pre-pandemic experience?

- A lot more excessive
- About the same amount
- Less workload
- I started my residency program during/after the pandemic

14. Do you ever have to work beyond your rostered hours or have to fill extra shifts?

- Always
- Usually
- Sometimes
- Rarely
- Never

15. Which of the following activities do you consider to contribute most to your workload? Order from 1-5, where 1 corresponds to contributing the most and 5 to contributing least

- [ ] On call shifts
- [ ] Scientific work - publications and posters
- [ ] Extra-shifts
- [ ] Postgraduate qualifications (i.e. PhD, Masters)
- [ ] Exams

16. I receive an adequate amount of training and supervision.

- Strongly agree
- Agree
- Neither agree or disagree
- Disagree
- Strongly disagree

RESIDENCY DURING THE COVID-19 PANDEMIC

17. Were you in an anaesthesiology residency program during the COVID-19 pandemic?

- ◯ Yes
- ◯ No

RESIDENCY DURING THE COVID-19 PANDEMIC

As you stated that you worked during the COVID-19 pandemic, please answer the following questions:

18. Were you moved to a different department as part of your residency during the COVID-19 outbreak?

- ◯ Yes, I was redeployed to a different department as part of my rotation
- ◯ No, I remained in my original department throughout the pandemic

19. Were you reassigned to the ICU during the COVID-19 pandemic?

- ◯ Yes, I was reassigned to work in the ICU during the pandemic
- ◯ No, I did not work in the ICU during the pandemic
- ◯ I was already working in the ICU during the pandemic

20. Did the COVID-19 pandemic impact your residency time, resulting in insufficient practical or surgical procedures for your level of training?

- ◯ Yes, my training was impacted by the pandemic and I did not receive enough practical or surgical procedures for my level of training
- ◯ No, my training was not significantly impacted by the pandemic

21. Have you been given an opportunity to make up for lost practical or surgical procedures due to the pandemic?

- ◯ Yes, I have been given opportunities to make up for lost practical or surgical procedures due to the pandemic
- ◯ No, I have not been given any opportunities to make up for lost training time
- ◯ I did not need to

22. Has your residency program been extended as a result of the COVID-19 pandemic?

- ◯ Yes, my residency program has been extended due to the pandemic
- ◯ No, my residency program has not been extended as a result of the pandemic

RESIDENCY COSTS

23. Please use the scale to express how significantly financial considerations might influence your participation in the following activities:

	Minimal Impact	Minor Impact	Moderate Impact	Major Impact	Extreme Impact
Attendance at conferences	○	○	○	○	○
Courses	○	○	○	○	○
Access to books and journals	○	○	○	○	○
Membership for scientific societies	○	○	○	○	○

24. Does your employer provide financial assistance when you are presenting research at a conference or attending further educational events?

- ○ Yes, Full Support: My employer fully covers the costs.
- ○ Partial Support: My employer covers some, but not all, of the costs.
- ○ Occasional Support: My employer occasionally provides financial support.
- ○ Rarely: My employer rarely provides financial support.
- ○ No Support: My employer does not provide any financial support.

25. Did you go to the Euroanaesthesia conference that took place in Glasgow in 2023?

- ○ Yes
- ○ No

26. If you did attend the Euroanaesthesia conference in Glasgow in 2023, what was your reason for attending?

You can choose multiple answers from the following options:

- [ ] To showcase and promote my scientific work
- [ ] To gain knowledge about the latest trends in the profession
- [ ] To network and meet other colleagues
- [ ] To explore new technologies in an industrial section
- [ ] To search for potential job opportunities
- [ ] Other (please specify)

27. Which of your expenses for Euroanaesathesia are being covered by your hospital/employer? You can select multiple options from the following:

- ○ None, I paid for everything myself
- ○ The hospital covers the cost of attendance fees
- ○ The hospital covers accommodation arrangements
- ○ The hospital covers transportation arrangements
- ○ The hospital covers all expenses

28. In the event that you did not attend the Euroanaesthesia Congress in Glasgow in 2023, what was the reason for your absence?

Please select one or more of the following options:

- ◯ The financial expenses were too high, and I had to cover them entirely myself
- ◯ The financial expenses were still too high, even with financial assistance from my hospital or employer
- ◯ I was unable to obtain paid leave from my hospital or employer
- ◯ I did not find the conference beneficial for my professional growth
- ◯ Other (please specify)

SUPPORT AND WELLBEING AT THE WORKPLACE

29. In regards to wellbeing, do you feel that you receive sufficient support at work?

- ◯ Yes
- ◯ No

30. Do you have access to any of the following support services?

- ☐ Coaching
- ☐ Psychological assistance
- ☐ Mentored support
- ☐ Non-medical support
- ☐ Counselling
- ☐ Other (please specify)

EXCHANGE PROGRAMS

31. During your residency, have you ever participated in training or exchange programs outside of your home country?

- ◯ Yes
- ◯ No

32. Why did you choose to partake in a **training or exchange program** outside of your home country?

- [ ] Skill Development at a Reference Center: You went abroad to acquire a specific skill at a renowned institution.
- [ ] Life Experience: You went abroad primarily for the personal experience and exposure to a new culture and environment.
- [ ] Institutional Request: Your home institution requested or required that you go abroad for training.
- [ ] Other (please specify)

33. Why did you choose **not** to partake in a **training or exchange program** outside of your home country?

- [ ] Costs: Financial constraints made it unfeasible for you to train abroad
- [ ] No Interest: You did not have the desire to participate in international training
- [ ] No Local Authorization: Your home institution or local authorities did not approve or allow international training
- [ ] Family: Personal or family responsibilities prevented you from training abroad
- [ ] Other (please specify)

EMPLOYMENT PROSPECTS

34. Are you contemplating relocating to another country?

- ◯ Yes
- ◯ No

If you answered 'Yes', please answer the following two questions:

35. What is your primary reason for considering emigration? (Please select one):

- ☐ Economic Reasons
- ☐ Desire for New Life Experiences
- ☐ Seeking Better Work-Life Balance
- ☐ Career Advancement Opportunities
- ☐ Returning to Home Country
- ☐ Family Reasons
- ☐ Other (please specify)

____

36. Which region are you considering for your emigration? (Please select one or provide a specific country)

- ☐ Northern Europe
- ☐ Western Europe
- ☐ Central Europe
- ☐ Eastern Europe
- ☐ Southern Europe
- ☐ South America
- ☐ North America
- ☐ Asia
- ☐ Australia
- ☐ Other (please specify)

____

CONCLUSION

37. Which of some of the previously explored issues do you consider most important to **improve**, as one of the **next initiative** of the **ESAIC Trainee Network**?
Order from 1-5, where 1 corresponds to the less important and 5 to the most important issue to improve.

- [ ] Exam/educational support
- [ ] Employment prospects
- [ ] Exchange programs
- [ ] Workload and supervision
- [ ] Resident wellbeing

38. Any further comments or feedback:
